# Supplementary material for: Inhibition of Bacterial Efflux Pumps by Crude Extracts and Essential Oil from Myristica fragrans Houtt. (Nutmeg) Seeds against Methicillin-Resistant Staphylococcus aureus
Source: Molecules. 2021 Jul 31;26(15):4662. doi: 10.3390/molecules26154662 (PMC8348620; doi:10.3390/molecules26154662)
Supplement: Supplementary file 1 [file molecules-26-04662-s001.zip › molecules-1297665-supplementary.pdf]

Supplementary data

Table S1. GCMS analysis of nutmeg crude extract.

No.	Peak area (%)	Peak name	RT (min)	Area	%Prob
1	22.663	Elemicin	33.028	3.97E+08	37.100
2	15.234	Tetradecanoic acid	40.898	2.67E+08	50.300
3	11.183	Myristicin	31.564	1.96E+08	73.800
4	6.880	4-Terpineol	16.724	1.20E+08	48.200
5	5.771	Sabinene	8.046	1.01E+08	26.300
6	2.774	Methoxyeugenol	34.719	4.85E+07	90.200
7	2.740	4-Thujanol, cis	13.158	4.80E+07	28.900
8	2.603	Decanoic acid, 2-oxo-, methyl ester	48.064	4.56E+07	12.500
9	2.283	Safrene	21.682	4.00E+07	38.900
10	2.283	n-Hexadecanoic acid	46.120	4.00E+07	45.900
11	2.238	4-Thujanol	11.802	3.92E+07	56.200
12	1.826	Isoelemicin	36.483	3.20E+07	66.500
13	1.746	Isoeugenol	28.603	3.06E+07	18.200
14	1.709	beta-Pinene	8.158	2.99E+07	17.400
15	1.446	Methyleugenol	26.744	2.53E+07	55.000
16	1.274	gamma-Terpinene	11.435	2.23E+07	34.000
17	1.161	alpha-thujene	6.461	2.03E+07	32.300
18	1.115	beta-Phellandrene	10.173	1.95E+07	25.900
19	0.958	alpha-Pinene	6.679	1.68E+07	16.500
20	0.909	Oleic Acid	48.716	1.59E+07	11.300
21	0.830	Octadecanoic acid, 3-oxo-, methyl ester	50.636	1.45E+07	20.800
22	0.798	alpha-Terpineol	17.315	1.40E+07	25.500
23	0.782	Benzoic acid, 2-acetyl-3-methoxy-	57.869	1.37E+07	22.300
24	0.758	2-Carene	9.674	1.33E+07	18.200
25	0.699	Cyclopentaneundecanoic acid	53.573	1.22E+07	18.400
26	0.642	beta-Caryophyllene	27.225	1.12E+07	36.300
27	0.589	Dodecanoic acid	33.414	1.03E+07	54.500
28	0.562	alpha-Terpineol acetate	24.305	9.83E+06	46.700
29	0.559	Geraniol acetate	25.807	9.79E+06	61.400
30	0.554	Isoeugenol methyl ether	30.568	9.70E+06	28.400
31	0.515	Copaene	25.404	9.02E+06	18.600
32	0.466	Terpinolene	12.704	8.15E+06	13.500
33	0.436	beta-Myrcene	8.672	7.63E+06	69.400
34	0.392	beta-Cymene	9.993	6.85E+06	23.500
35	0.380	trans-Pinene hydrate	14.168	6.65E+06	35.600
36	0.350	Linalool	13.243	6.12E+06	36.500
37	0.307	2-(3-Methylphenyl)-2-propanol	17.095	5.37E+06	41.400
38	0.260	Hedycaryol	32.509	4.54E+06	26.400
39	0.222	4-Carene, (1S,3S,6R)-(-)-	9.421	3.88E+06	14.300
40	0.221	beta-Cubebene	29.762	3.87E+06	19.300
41	0.213	Citronellol acetate	24.495	3.73E+06	32.400
42	0.197	o-Eugenol	24.728	3.45E+06	17.500
43	0.190	Isobornyl acetate	21.545	3.33E+06	25.200
44	0.166	alpha-Fellandrene	9.188	2.91E+06	36.200
45	0.161	trans-Piperitol	18.096	2.82E+06	29.100

Table S2. GCMS analysis of nutmeg essential oil.

No.	Peak area (%)	Peak name	RT (min)	Area
1	36.907	Sabinene	11.726	5.6E+09
2	11.544	4-Terpineol	21.444	1.8E+09
3	9.414	alpha-Pinene	9.760	1.4E+09
4	6.135	beta-Phellandrene	14.149	9.4E+08
5	4.445	(-)-BETA-PINEN	11.784	6.8E+08
6	3.312	gamma-Terpinene	15.575	5.1E+08
7	2.624	beta-Myrcene	12.415	4.0E+08
8	2.551	Myristicin	36.323	3.9E+08
9	2.549	cis-Asarone	37.741	3.9E+08
10	1.884	1,3-Benzodioxole, 5-(2-propenyl)	26.392	2.9E+08
11	1.826	alpha-Terpinene	13.504	2.8E+08
12	1.774	alpha-Thujene	9.433	2.7E+08
13	1.690	m-Cymene	13.910	2.6E+08
14	1.586	3-Carene	13.175	2.4E+08
15	1.464	Terpinolene	16.937	2.2E+08
16	1.372	Tetradecanoic acid	45.666	2.1E+08
17	1.002	alpha-Terpineol	21.962	1.5E+08
18	0.944	Methyleugenol	31.507	1.4E+08
19	0.781	4-THUJANOL, STEREOISOMER	15.980	1.2E+08
20	0.733	alpha-Phellandrene	12.897	1.1E+08
21	0.710	p-Menth-2-en-1-ol, trans	18.571	1.1E+08
22	0.710	4-THUJANOL, CIS-(.+-.)-	17.466	1.1E+08
23	0.573	beta-Linalool	17.641	8.8E+07
24	0.452	p-Menth-2-en-1-ol, cis	19.487	6.9E+07
25	0.424	Geraniol acetate	30.481	6.5E+07
26	0.252	Citronellol acetate	29.162	3.9E+07
27	0.252	trans-Piperitol	22.705	3.9E+07
28	0.241	alpha-Terpineol acetate	28.945	3.7E+07
29	0.199	Copaene	30.016	3.1E+07
30	0.190	Borneol acetate	26.124	2.9E+07
31	0.181	Isoeugenol methyl ether	35.312	2.8E+07
32	0.165	cis-Piperitol	22.112	2.5E+07
33	0.153	Camphene	10.275	2.3E+07
34	0.142	Isoelemicin	41.186	2.2E+07
35	0.113	Cuminol	21.768	1.7E+07
36	0.113	trans-alpha-Bergamotene	32.559	1.7E+07
37	0.095	Germacrene D	34.392	1.5E+07
38	0.089	trans-Geraniol	24.904	1.4E+07
39	0.082	TRANS-SABINENE HYDRATE ACETATE	26.790	1.3E+07
40	0.081	Farnesol	35.553	1.2E+07
41	0.079	Caryophyllene	31.837	1.2E+07
42	0.075	beta-Citronellol	23.719	1.1E+07
43	0.048	Dodecanoic acid	38.195	7.3E+06
44	0.045	Eugenol	29.414	7.0E+06

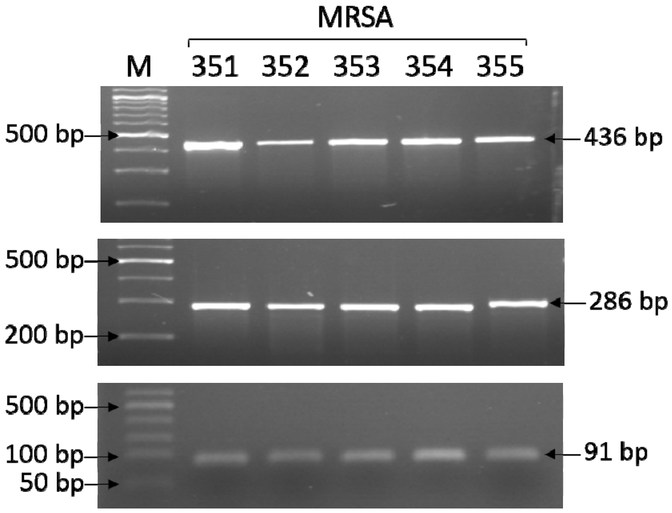

Figure S1. Agarose gel electrophoresis of five MRSA isolates. Identification of resistant strains was performed by detection of *mec*A gene (middle, 286 bp), and identification of efflux pumps were performed by detection of *nor*A (upper, 436 bp) and *mep*A (lower, 91 bp) genes in all MRSA strains using PCR assay. Marker (M) and MRSA351-355 strains, respectively.

Supplementary materials and methods

1. Total Phenolic content

Total Phenolic content (TPC) was determined using a modified method of Singleton V. L. and Rossi J.A. in 1965. The aqueous sample solution with concentration of 0-100 µg/mL was prepared using a 96-well plate model, the reaction mixture that comprised 50 µL of sample solution, 50 µL of 50% v/v Folin-Ciocalteu reagent and 125 µL of 20% w/v sodium carbonate was constituted and mixed gently. Following 40 min incubation at room temperature, the optical absorbance was determined at 700 nm using a microplate reader (Sunrise™ micro plate Reader, Männedorf, Switzerland) standard curve development (Gallic acid). TPC was expressed as mg gallic acid equivalence (mg GAE eq/g extract).

2. Total Flavonoid content

Total flavonoid content (TFC) was determined using a modified method of Chang, Zuo, Harrison and Chow in 2002. The sample aqueous solution with concentration of 0-100 µg/mL was prepared using a 96-well plate model, the reaction mixture that comprised 100 µL of sample solution, 100 µL of 2%(w/v) AlCl3 solution was constituted and mixed gently. Following 30 min incubation at room temperature, the optical absorbance was determined at wavelength of 437 nm using microplate reader (Sunrise™ micro plate Reader, Männedorf, Switzerland) standard curve development (Quercetin). TFC was expressed as quercetin equivalence (mg QE eq/g extract).
